# A risk-gate mechanistic–ecological framework for assessing emergence potential of henipa-related viruses

**DOI:** 10.3389/fmicb.2026.1827085

**Published:** 2026-06-18

**Authors:** Wenpeng Tian, Hong Xiao, Yongxia Gao, Mingming Liu, Guangyu Qiu

**Affiliations:** 1 Department of Emergency, Xiangyang Central Hospital, Affiliated Hospital of Hubei University of Arts and Science, Xiangyang, China; 2 School of Basic Medicine, Hubei University of Arts and Science, Xiangyang, China; 3 Nursing Department, Xiangyang Central Hospital, Affiliated Hospital of Hubei University of Arts and Science, Xiangyang, China

**Keywords:** Hendra virus, Langya virus, Mojiang virus, Nipah virus, One Health, risk-gate framework

## Abstract

Henipa-related viruses can cause severe disease following rare zoonotic introductions, yet spillover alone does not determine the likelihood of outbreak emergence. Here we propose a risk-gate mechanistic–ecological framework to assess emergence potential across henipa-related viruses. Using the well-characterized henipaviruses Nipah and Hendra together with the more recently described Langya and Mojiang viruses as comparative examples, we define a sequential pathway linking upstream exposure (Step 0) to host entry and tissue access (Gate 1), within-host replication and immune antagonism (Gate 2), and transmission amplification (Gate 3). By tagging the strength of available evidence and identifying gate-specific knowledge gaps, the framework distinguishes biological constraints from operational uncertainties including diagnostics, infection prevention and control (IPC), and response capacity. This evidence-tagged framework provides a practical One Health roadmap for comparing established and emerging henipa-related viral threats. We present it as a conceptual, review-oriented tool rather than a validated predictive model and outline how future retrospective outbreak analyses could test its utility.

## Introduction

Zoonotic emergence is often framed as a single moment of “spillover.” For henipaviruses and related parahenipaviruses, however, public health outcomes reflect a sequence of biological and ecological constraints, including exposure, host entry, within-host fitness, and transmission amplification (Zhou et al., 2020; Quarleri et al., 2022; Wang L. et al., 2024; Spengler et al., 2025). Focusing on risk assessment solely at the spillover step can obscure why similar bat–human or animal–human interfaces sometimes result in silent dead ends and other times generate explosive, health care-associated clusters (Middleton et al., 2014; Plowright et al., 2016; Rissanen et al., 2017; Yuen et al., 2021). This hypothesis and theory article argues that “spillover is not enough”: while upstream exposure creates opportunity, human impact depends on whether successive risk gates are crossed within real-world contact networks (Eaton et al., 2006; Halpin and Rota, 2015; Weatherman et al., 2018; Piracha et al., 2023). We integrate mechanistic virology with ecological context into a practical framework to prioritize evidence gaps and countermeasure development. Here, mechanistic virology includes entry receptors, innate immune antagonism, and tissue tropism, whereas ecological context includes reservoir dynamics, intermediate hosts, human behavior, and care settings (Halpin and Mungall, 2007; Xu et al., 2012a; Navaratnarajah et al., 2020; Kadir et al., 2023; Wu et al., 2024; Li J. D. et al., 2025).

Classical bat-borne henipaviruses—Nipah virus (NiV) and Hendra virus (HeV)—show that a virus can have high intrinsic severity while producing very different transmission outcomes across places and time (Bardhan et al., 2023; Veggalam et al., 2025; World Health Organization, 2026a). In contrast, recently described parahenipaviruses (e.g., Langya virus, LayV; Mojiang virus, MojV) sit in the broader clade yet leave key risk gates unresolved, particularly receptor biology and the status of human infection or onward transmission (Shariff, 2019; Dong et al., 2020; He et al., 2024). Rather than providing a descriptive overview alone, this article develops an evidence-tagged, gate-mapped synthesis to identify what is confirmed, what is supported, and what remains unresolved across NiV, HeV, LayV, and MojV. We also apply the framework against a current event: the 2026 NiV reports from West Bengal, India. Early official updates describe few cases and negative testing among traced contacts, yet the episode illustrates how amplification factors—care settings, close-contact patterns, and response speed—can act as decisive “accelerators” even when the detected spread remains limited (Eaton et al., 2006; Halpin and Rota, 2015; Australian Centre for Disease Control, 2026; European Centre for Disease Prevention and Control, 2026).

## Scope, terminology, and evidence grading

### Scope

We focus on two henipaviruses with confirmed human disease (NiV and HeV) and two parahenipaviruses that sharpen mechanistic uncertainty (LayV and MojV) (Negrete et al., 2006; Van Doremalen et al., 2022; Frenck et al., 2025; Shafaati and Zandi, 2025). The comparison is intentional: NiV and HeV are used as better-characterized anchor examples, whereas LayV and MojV are included to expose unresolved gates, especially receptor use, tissue permissiveness, human infection status, and the limits of extrapolation (Halpin et al., 2021; Mallapaty, 2022; Hejran et al., 2025; National Centre for Disease Control, 2026; Press Information Bureau, Government of India, 2026).

### Terminology

We use “Exposure” for upstream contact opportunities (reservoir, intermediate host, contaminated food, occupational or healthcare contact). “Entry” refers to cellular compatibility and tissue access. “Within-host fitness” covers replication and immune antagonism sufficient to sustain infection and establish relevant tropism. “Amplification” refers to within- and between-host processes that increase onward transmission probability (viral shedding, contact-network structure, and amplification hosts) (Plowright et al., 2016; Weatherman et al., 2018).

### Evidence grading

To keep claims traceable, we apply the evidence tags defined in Table 1. Confirmed denotes direct experimental demonstration or repeated field confirmation. Supported denotes convergent but incomplete evidence, for example *in vitro* permissiveness without *in vivo* confirmation. Unknown denotes a lack of decisive data or genuinely conflicting reports. We assign tags by gate rather than by virus overall because evidence strength differs across domains (Guyatt et al., 2008; Borisevich et al., 2015; Halpin and Rota, 2015). These labels indicate evidentiary status rather than formal study quality. Where data are sparse or conflicting, we use the more conservative label and state the uncertainty explicitly.

**Table 1:** Evidence-tagged, operationalized: review-oriented outputs from the Risk-Gate framework.

Risk-Gate domain (what must be “passed”)	NiV (classical bat-borne)	HeV (classical bat-borne)	LayV (small-mammal-associated)	MojV (small-mammal-associated)	Priority review-oriented outputs	References
Gate 1: Cellular compatibility (receptor usage and entry constraints)	[Confirmed] Uses ephrin-B2/B3 for entry; broad host/tissue permissiveness consistent with cross-species transmission	[Confirmed] Uses ephrin-B2/B3 for entry; spillover risk shaped more by exposure context than entry alone	[Supported] Does not bind ephrin-B2/B3; [Unknown] receptor unresolved; key uncertainty is which human/host tissues are permissive	[Supported] Does not use ephrin-B2/B3; attachment glycoprotein suggests a distinct entry pathway; [Unknown] receptor(s) unknown; whether entry supports human infection remains uncertain	Entry evidence map (Confirmed/Supported/Unknown) + likely bottlenecks	Negrete et al. (2006), Bishop et al. (2007), Xu et al. (2012a,b), Rissanen et al. (2017), Navaratnarajah et al. (2020), Cheliout Da Silva et al. (2021), World Health Organization (2022a,b), Zhang et al. (2022), Wang C. et al. (2024), Wang L. et al. (2024)
Gate 2: Within-host fitness (IFN antagonism, replication, tropism)	[Confirmed] Strong IFN antagonism (e.g., V/W/C proteins); supports severe neuro-respiratory disease potential	[Supported] Similar IFN antagonism; severe disease documented, but within-host dynamics less comprehensively characterized	[Unknown] Within-host determinants poorly defined; [Supported] current evidence suggests mostly mild–moderate illness; gaps include neuroinvasive potential and host factors shaping severity	[Unknown] Human within-host data absent/insufficient; gaps include replication competence in human-relevant systems, immunopathology, and clinical phenotype	Severity mechanism shortlist + evidence grading + research priorities	Rodriguez et al. (2003), Shaw et al. (2004), Misako et al. (2010), Wu et al. (2014), Zhang et al. (2022), Paliwal et al. (2024)
Gate 3: Transmission gate (amplification hosts, exposure networks, human → human spread)	[Confirmed] Multiple spillover routes (food-borne/date palm sap; intermediate hosts); [Confirmed] human → human transmission in some outbreaks	[Confirmed] Spillover chain: bats → horses → humans; [Confirmed] no sustained human → human transmission; risk concentrated in horse-associated exposures	[Unknown] Zoonotic spillover plausible; exposure interface still being clarified; [Supported] no sustained human → human transmission observed to date	[Confirmed] Identified in surveillance with rodent association; [Unknown] amplification/transmission chain unverified; no confirmed human infections	Exposure–amplification hypothesis set + risk-upgrading evidence triggers	Gurley et al. (2007), Khan et al. (2012), Wu et al. (2014), Zhang et al. (2022), Paliwal et al. (2024)
Cross-cutting: Surveillance and interpretation	[Supported] Integrate ecological signals with clinical surveillance to anticipate outbreak windows	[Supported] Strengthen the horse → human One Health interface and maintain routine monitoring	[Supported] Standardize case definitions and surveillance triggers to reduce uncertainty	[Supported] Prioritize confirming/refuting human infection: high-quality serology + syndromic surveillance + ecological evidence	Evidence-grading framework + early-warning trigger list	Hassan et al. (2023), Paliwal et al. (2024), Branda et al. (2025)

Key evidence gaps and priority research questions to operationalize the Risk-Gate framework for representative henipaviruses and parahenipaviruses.

### Pragmatic implications

We avoid the common leap from upstream mechanistic evidence (cell entry; within-host replication and immune antagonism) to downstream epidemiological conclusions (transmission amplification or sustained human-to-human spread) (Chakraborty et al., 2022; Haas and Lee, 2023; Branda et al., 2024). Likewise, frequent exposure alone does not imply cellular compatibility. When we rely on analogies (e.g., broader paramyxovirus patterns), we explicitly label such statements as Supported or Unknown (Li et al., 2020; Qiu et al., 2024; Rahim et al., 2024; Hantabal et al., 2026).

## Geographic and phylogenetic context

**Figure 1:**
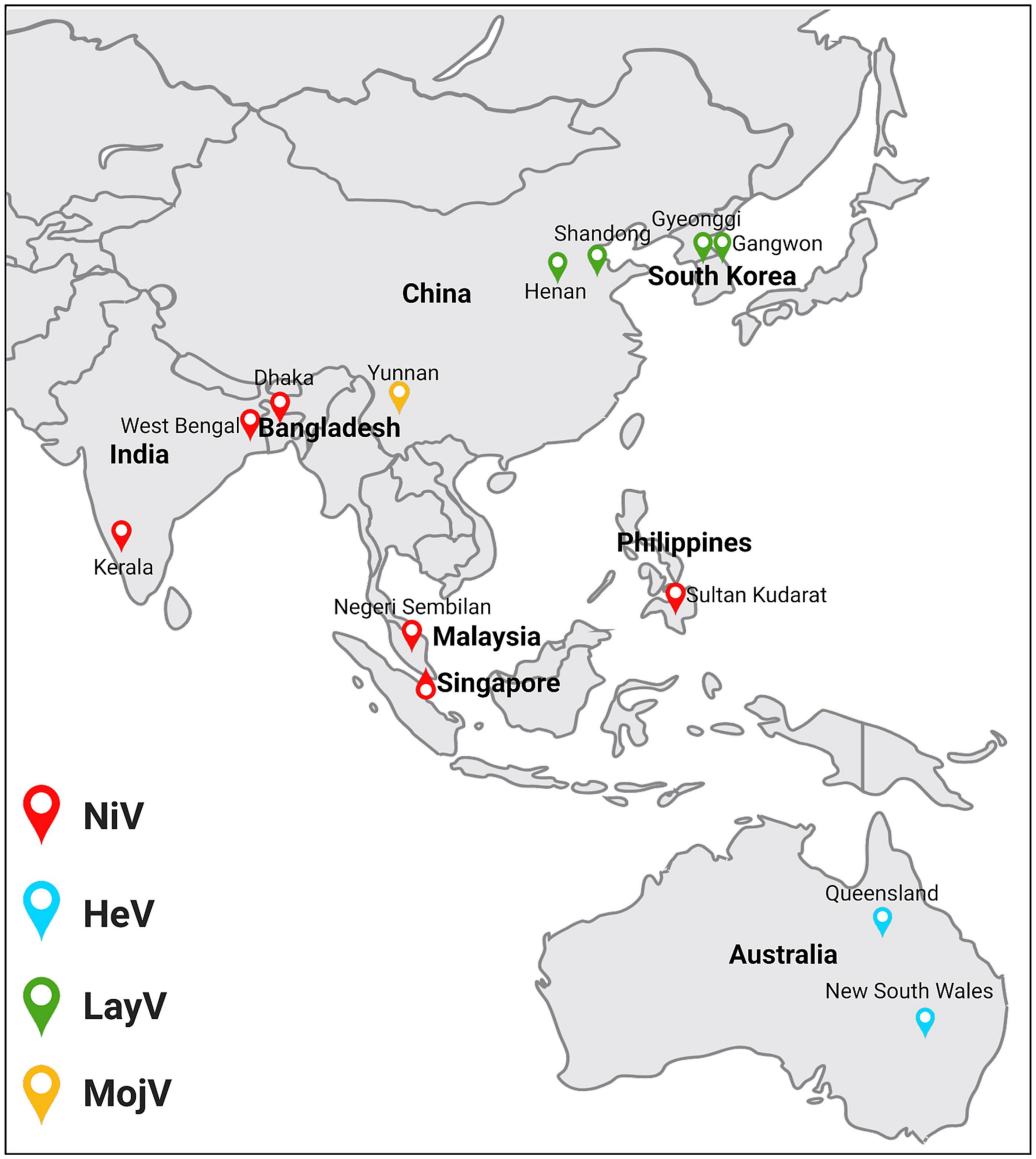
Reported geographic evidence for representative henipaviruses and parahenipaviruses. Human infections have been reported for NiV in Bangladesh (Dhaka Division), India (West Bengal and Kerala), Malaysia (Negeri Sembilan), the Philippines (Sultan Kudarat), and Singapore; for HeV in Australia (Queensland and New South Wales); and for LayV in China (Shandong and Henan Provinces). LayV has also been detected through *Crocidura lasiura* surveillance in South Korea (Gyeonggi and Gangwon Provinces), but this does not constitute confirmed human infection in South Korea. MojV has been detected as viral RNA in *Rattus flavipectus* from Mojiang Hani Autonomous County, Yunnan Province, China, with human infection remaining unconfirmed. This figure is intended as a geographic evidence map rather than a vector-range or quantitative risk map. Geographic boundaries are schematic and used only for visualization. Owing to Singapore’s small land area, its NiV marker denotes both the reported location and the country-level evidence, with the Malaysia–Singapore boundary highlighted for clarity.

Geography and phylogeny are used here as context for evidence interpretation, not as direct proxies for emergence potential (Figure 1; Table 2) (Plowright et al., 2016; Quarleri et al., 2022; Li et al., 2023; Tan et al., 2024). A comparative overview of key virological and epidemiological features of NiV, HeV, LayV, and MojV is provided in Table 2. Human disease has been confirmed for NiV and HeV in distinct geographic and ecological settings, and those settings shape both exposure and amplification opportunities (Field et al., 2010; Li et al., 2023; Wang L. et al., 2024). NiV has been associated with multiple event types: food-borne spillover linked to bat-contaminated products (notably date palm sap in Bangladesh), direct animal contact (e.g., pigs in Malaysia/Singapore), and healthcare-associated amplification with person-to-person transmission in some outbreaks (Luby et al., 2009; Khan et al., 2012). Taken together, these events show that the exposure route and contact structure are part of the causal chain, not background context (Luby et al., 2009; Weatherman et al., 2018). HeV-related human disease has occurred mainly through bat-to-horse-to-human spillover, concentrating risk in occupational settings involving close horse contact and animal care. This creates a different “exposure funnel” and a different amplification profile than NiV, despite similar Gate 1–2 biology (Eaton et al., 2006; Peel et al., 2019; Halpin et al., 2021; Rahim et al., 2024). As a real-time anchor, official reports in early 2026 described two confirmed NiV cases in West Bengal and extensive contact testing, with no additional positives cases, a pattern consistent with upstream exposure and care-setting risk but constrained Gate 3 amplification at the time of reporting (European Centre for Disease Prevention and Control, 2026; Press Information Bureau, Government of India, 2026; World Health Organization, 2026a).

**Table 2:** Comparative overview of key virological and epidemiological features of representative henipaviruses and parahenipaviruses: Nipah virus (NiV), Hendra virus (HeV), Langya virus (LayV), and Mojiang virus (MojV).

Characteristic	Nipah virus (NiV)	Hendra virus (HeV)	Langya virus (LayV)	Mojiang virus (MojV)	References
Virus group	Classical bat-borne	Classical bat-borne	Emerging parahenipavirus	Emerging parahenipavirus	Eaton et al. (2006), Li et al. (2023), Satter et al. (2025)
Genome length (−ssRNA)	18,246 nt	18,234 nt	18,402 nt	18,404 nt	Wang et al. (2000), Eaton et al. (2006), Piracha et al. (2023), Satter et al. (2025)
Core gene order	N–P–M–F–G–L	N–P–M–F–G–L	N–P–M–F–G–L	N–P–M–F–G–L	Eaton et al. (2006), Satter et al. (2025), Spengler et al. (2025)
Natural reservoir host	Pteropus fruit bats	Pteropus fruit bats	Shrews (*Crocidura* spp.)	Rodents (*Rattus flavipectus*)	Peel et al. (2019), Satter et al. (2025), Spengler et al. (2025)
Intermediate/amplifier host(s)	Pigs (amplifier; Malaysia outbreak)	Horses (main spillover host)	Unknown; possible domestic exposure interface	Unknown	Luby et al. (2009), Field et al. (2010), Satter et al. (2025), Spengler et al. (2025)
Main spillover pathways to humans	Bat contamination of food; raw date palm sap; pig-mediated spillover	Bat → horse → human contact	Likely animal → human spillover (route unresolved)	Unconfirmed zoonotic transmission	Luby et al. (2009), Field et al. (2010), Satter et al. (2025), Spengler et al. (2025)
Human-to-human transmission	Reported (notably in South Asia outbreaks)	N/A	N/A	N/A	Luby et al. (2009), Field et al. (2010), Satter et al. (2025), Spengler et al. (2025)
Typical human disease	Encephalitis ± severe respiratory illness	Severe respiratory and neurologic disease	Febrile illness; mostly mild–moderate	Unconfirmed human disease	Quarleri et al. (2022), Satter et al. (2025), Ali et al. (2026)
Human case fatality (reported)	40–75% (varies by outbreak)	57%	No deaths reported in initial cases	N/A	Luby et al. (2009), Satter et al. (2025), Spengler et al. (2025)
Geographic distribution (reported)	Bangladesh (Dhaka Division)/India (West Bengal, Kerala)/Malaysia (Negeri Sembilan)/Philippines (Sultan Kudarat)/Singapore	Australia (Queensland, New South Wales)	China (Shandong Province, Henan Province), South Korea (Gyeonggi Province, Gangwon Province)	China (Mojiang Hani Autonomous County, Yunnan Province)	Rissanen et al. (2017), Natasha et al. (2025), Rajkhowa et al. (2025), Satter et al. (2025), Spengler et al. (2025)
Zoonotic risk summary	High (major outbreaks + human → human)	High (high severity; horse → human)	Moderate (zoonotic spillover confirmed; severity lower so far)	Unknown (host and receptor biology distinct; human infection unclear)	Li et al. (2023), Satter et al. (2023, 2025), Bhowmik et al. (2025), Spengler et al. (2025), Tripp et al. (2025)

LayV, by contrast, has been detected in human cases in China in the context of small mammal–associated ecology, but the reservoir-to-human chain and the relevance of human-to-human spread remain limited or unconfirmed in the available reports (Chen and Quigley, 2022; Tabassum et al., 2022; Adesola et al., 2023; Uwishema et al., 2023). MojV has been identified in rodents, with incomplete evidence for human infection, making it useful as an “unknown-gate” comparator rather than an outbreak analog (Chakraborty et al., 2022; Mallapaty, 2022; He et al., 2024; Li W. et al., 2025). Geography should be interpreted as a map of exposure opportunities, not a map of inevitable emergence (Choudhary et al., 2022; World Health Organization, 2022b; Nayak, 2025; Public Health Ontario, 2026). Apparent quiescence versus cluster detection can shift with surveillance intensity, exposure seasonality, and the presence of amplification settings such as hospitals (Pryce et al., 2020; Annand et al., 2022; Pollak et al., 2023; Satter et al., 2023; Dunga et al., 2025; Natasha et al., 2025; Qiu et al., 2026).

**Figure 2:**
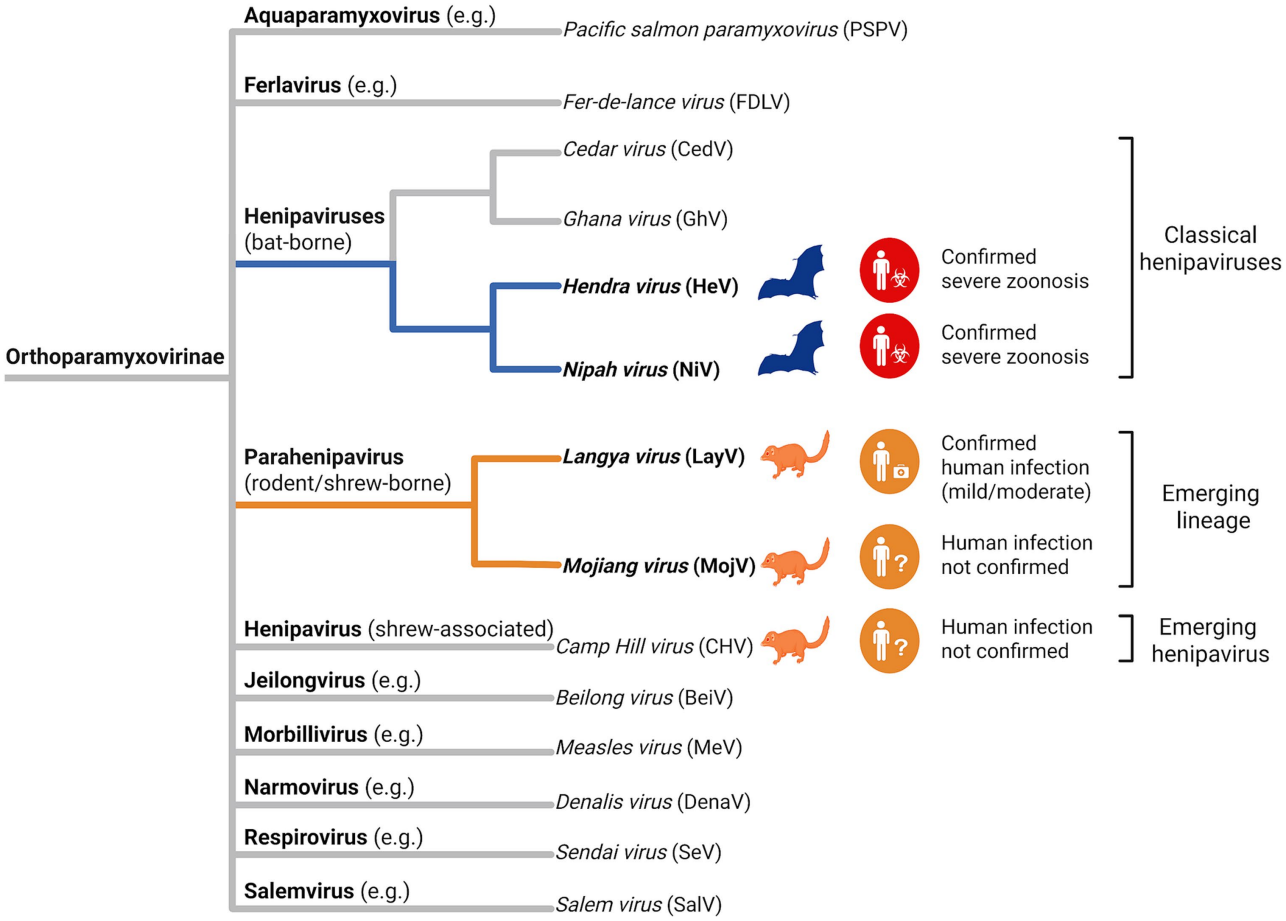
Phylogeny overview. Schematic taxonomic/phylogenetic overview of selected Orthoparamyxovirinae, contrasting classical bat-borne henipaviruses (Henipavirus; e.g., Nipah virus [NiV], Hendra virus [HeV]) with emerging lineages associated with rodents and shrews (Parahenipavirus; e.g., Langya virus [LayV], Mojiang virus [MojV]). Labels summarize current evidence for human infection and clinical impact (confirmed severe zoonosis for NiV/HeV; confirmed mild to moderate human infection for LayV; human infection not yet confirmed for MojV). Camp Hill virus is included as a shrew-associated henipa-related comparator, with no confirmed human infection to date.

Henipaviruses sit within Orthoparamyxovirinae and encompass lineages that differ markedly in host associations, receptor usage, and disease phenotypes (Haas and Lee, 2023; Tripp et al., 2025; Hantabal et al., 2026). The phylogenetic context is summarized to illustrate why “henipavirus-like” alone is a poor predictor of emergence risk without mechanistic annotation (Figure 2) (Xu et al., 2008; Li et al., 2024; Oguntuyo et al., 2024; Kane et al., 2025). Classic bat-borne henipaviruses (NiV and HeV) cluster around a shared entry logic (ephrin-B2/B3 usage) and a suite of P-gene products that shape innate immune antagonism (Wang et al., 2000; Xu et al., 2012b; Annand et al., 2022; Ahamed et al., 2026). These conserved features help explain the broad host range and severe neuro-respiratory disease potential once infection is established (Xu et al., 2008; Field et al., 2010). In contrast, parahenipaviruses (e.g., LayV and MojV) differ in host ecology and in key molecular interfaces, including lack of ephrin-B2/B3 usage in some members and incomplete mapping of attachment and fusion determinants (Cheliout Da Silva et al., 2021; Chavda et al., 2023; Carey et al., 2025; Chen et al., 2025a). That divergence makes them informative comparators: it helps distinguish gates that are untested from gates that are truly absent (Mohd-Qawiem et al., 2022; Caruso and Edwards, 2023; Kaza and Aguilar, 2023; Li Y. et al., 2025).

Taxonomy matters in practice because surveillance interpretation and countermeasure extrapolation hinge on genus- and species-level assumptions (Wang et al., 2000; Khandia et al., 2019; Yin et al., 2021). Vaccine antigen design, for example, may leverage conserved epitopes within NiV/HeV clades, whereas entry predictions for LayV/MojV depend on receptor identification before any confident host-range inference (Rissanen et al., 2017; Halpin et al., 2021; Frenck et al., 2025; Hejran et al., 2025). We therefore use phylogeny as a hypothesis generator, not as a substitute for evidence: relatedness can prioritize experiments (receptor binding, IFN antagonism assays, and tropism models), but it does not determine Gate 3 outcomes in human contact networks (Bishop et al., 2007; Dong et al., 2020; Navaratnarajah et al., 2020).

## Host range and spillover pathways

**Figure 3:**
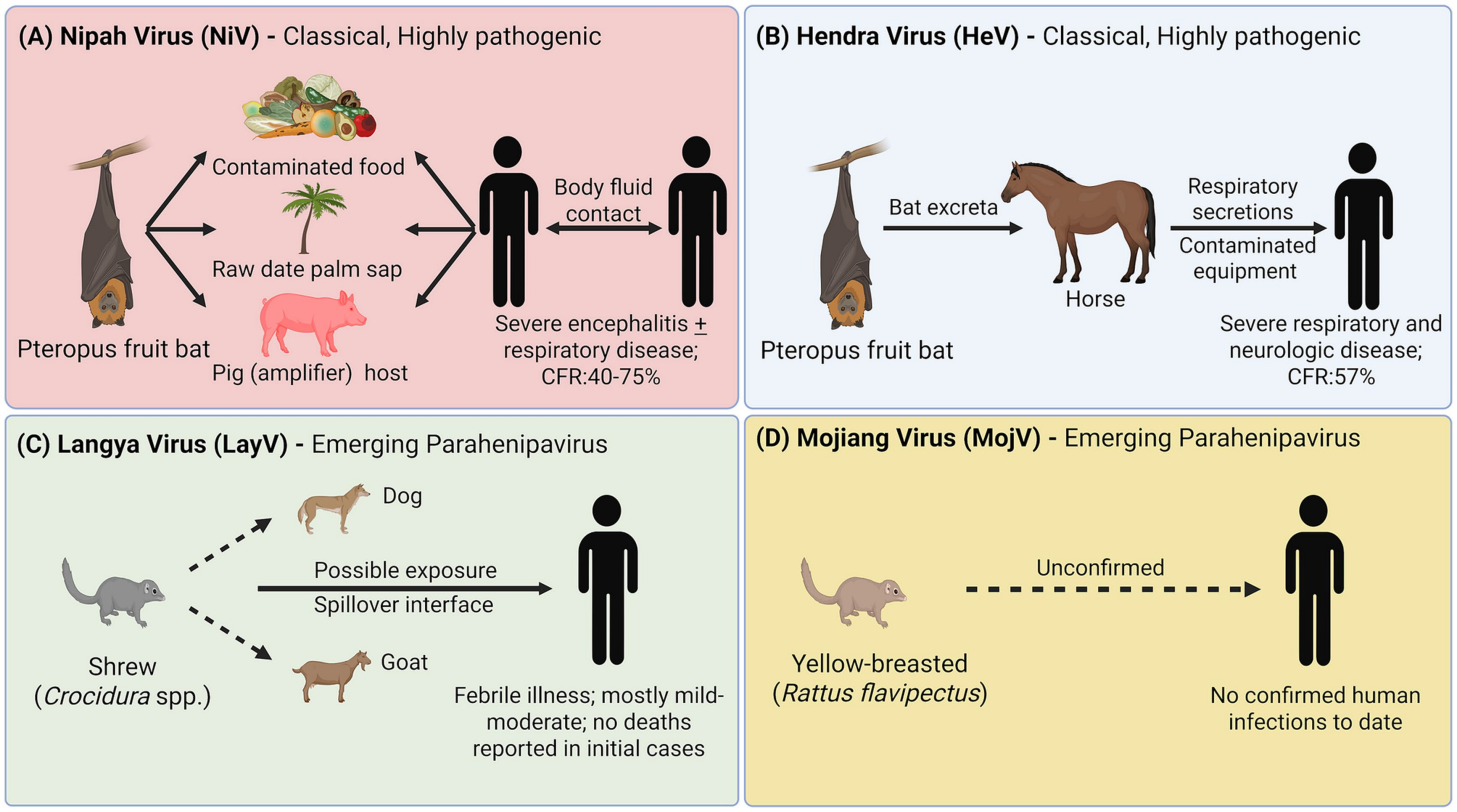
Host range and spillover pathways of representative henipaviruses and parahenipavirus. Overview of reservoir hosts, exposure routes, and clinical outcomes for four representative viruses. **(A)** NiV: spillover from *Pteropus* fruit bats via contaminated food or raw date palm sap, with pig amplification and subsequent close-contact transmission; associated with severe encephalitis and/or respiratory disease (reported CFR: 40–75%). **(B)** HeV: bat-to-horse spillover via excreta, with human infections linked to horse exposure through respiratory secretions or contaminated equipment (reported CFR: 57%). **(C)** LayV: shrew-associated virus with a suggested spillover interface and mostly mild to moderate febrile illness in initial human cases. **(D)** MojV: rodent-associated virus with no confirmed human infections to date; dashed arrows denote possible or unconfirmed links.

Host range reflects more than just entry and replication; it also depends on contact opportunity and interface ecology (Bhowmik et al., 2025; Nahar et al., 2025; Satter et al., 2025; Ali et al., 2026). With respect to NiV and HeV, *Pteropus* fruit bats are key reservoirs, but spillover pathways differ: NiV often involves food contamination and/or intermediate hosts (historically including pigs), whereas HeV follows a well-characterized bat–horse–human pathway (Rajkhowa et al., 2025). These routes are summarized, and the points where evidence is strongest are highlighted (Figure 3) (Peel et al., 2019; Choi, 2021; Satter et al., 2023).

Upstream exposure (Step 0) is shaped by reservoir shedding dynamics, seasonality, human behaviors (e.g., raw sap consumption), and husbandry practices that create bridging opportunities (Peel et al., 2019; Baranowski and Bharti, 2023; Haas and Lee, 2023). These determinants can often be targeted without invoking viral genetic change, making them actionable One Health levers even when mechanistic gate data are incomplete (Khan et al., 2012; Plowright et al., 2016; Yuen et al., 2021; Dunga et al., 2025). Intermediate or amplifier hosts can turn rare bat–human contacts into high-dose, high-frequency exposure networks. The Malaysia outbreak illustrates pig amplification, and HeV illustrates equine amplification; in both cases, amplifier-host ecology can dominate outbreak scale even when human-to-human transmission is absent or limited (Peel et al., 2019; Pollak et al., 2023). LayV and MojV raise a different set of ecological questions centered on small-mammal interfaces, occupational exposure (e.g., farming, animal contact), and surveillance within febrile/respiratory syndromes. For these viruses, the upstream gate is often most visible, whereas downstream gates remain mechanistically underdefined—an evidence gap that should be stated explicitly rather than filled by analogy (Hejran et al., 2025).

Across viruses, synthesis requires connecting exposure interfaces to plausible target tissues: the contact route (respiratory droplets, contaminated food, and animal secretions) should be mapped to the portal-of-entry cells that Gate 1 must reach. This conceptual bridge links Figure 3 (interfaces) to Gate 1 biology and helps keep ecological and mechanistic claims aligned (Rissanen et al., 2017; Navaratnarajah et al., 2020; Tripp et al., 2025).

## The risk-gate mechanistic–ecological framework

**Figure 4:**
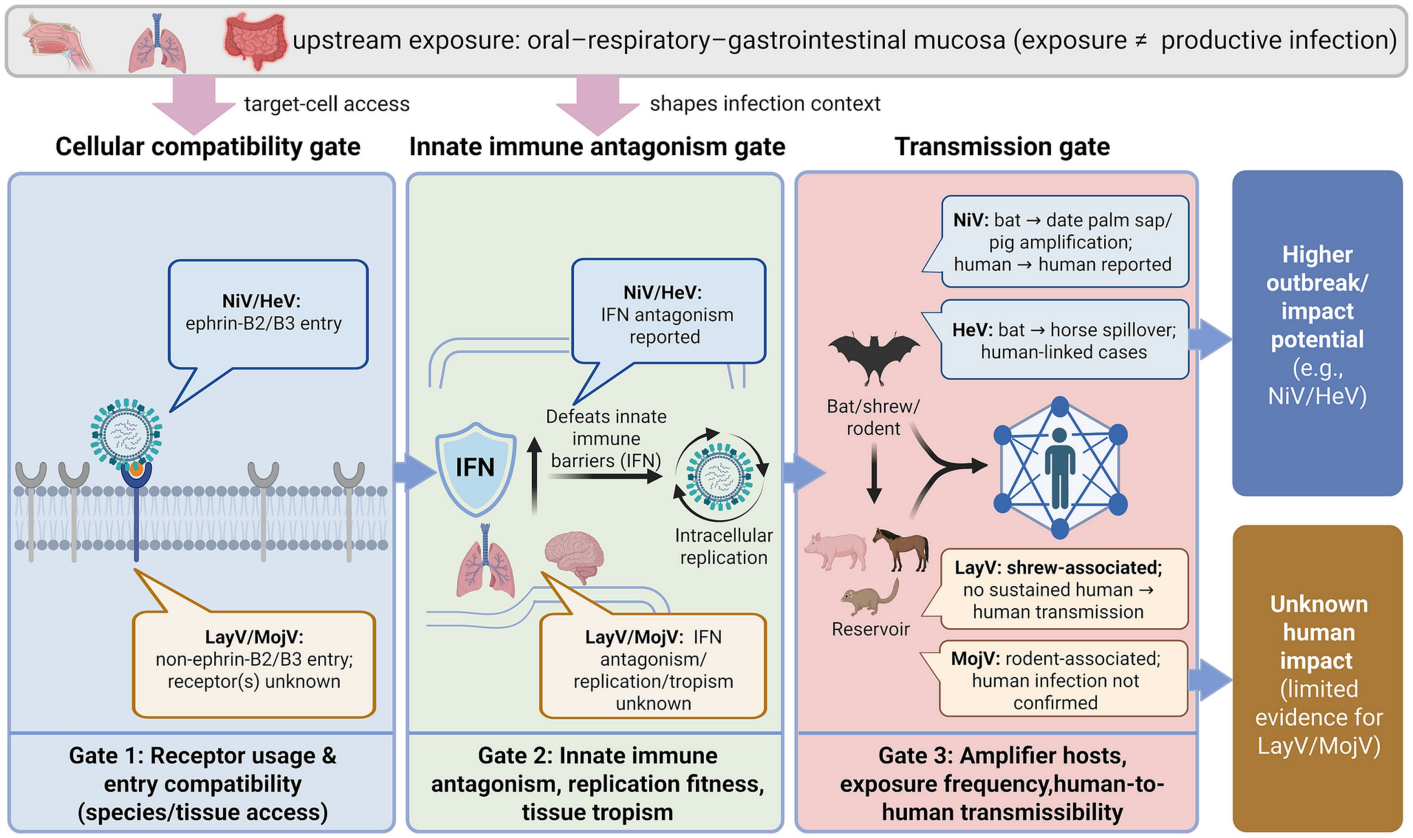
Risk-gate framework for zoonotic emergence of representative henipaviruses and parahenipavirus. Conceptual synthesis based on published evidence; not a *de novo* phylogenetic or quantitative risk model. Conceptual “risk-gate” model depicting how spillover opportunity translates into zoonotic impact through three sequential gates. Gate 1 captures receptor usage and entry compatibility (species and tissue access), Gate 2 summarizes innate immune antagonism and replication fitness/tissue tropism, and Gate 3 reflects transmission amplification via exposure frequency, amplifier hosts, and human-to-human transmissibility. Callouts contrast NiV/HeV (ephrin-B2/B3 entry; reported IFN antagonism via P gene products such as V/W/C) with LayV/MojV (do not bind ephrin-B2/B3; receptor(s) unknown; immune evasion/pathogenesis less characterized) and indicate higher outbreak potential for NiV/HeV versus currently limited or uncertain human impact for LayV/MojV.

The central contribution of this article is a risk-gate framework that separates upstream exposure from three gates—entry, within-host fitness, and amplification—and uses heterogeneous evidence to guide surveillance triggers, experimental priorities, and countermeasure design (Figure 4) (Halpin et al., 2021; Dunga et al., 2025; Mortlock et al., 2025). Gate 1 asks whether the virus can access and enter relevant human (or amplifier-host) target cells; Gate 2 asks whether replication and innate immune antagonism are sufficient to sustain infection with clinically and epidemiologically relevant tropism; Gate 3 asks whether real-world networks and shedding dynamics permit amplification—in animals or humans—that increases onward transmission probability (Eaton et al., 2006; Halpin and Rota, 2015; Weatherman et al., 2018). This logic is presented as a gate-based schematic, with parallel evidence tracks for well-characterized NiV/HeV and less-characterized LayV/MojV (Figure 4).

The framework also accommodates “unknown gates”: for LayV and MojV, exposure signals exist, but receptor usage and tissue permissiveness remain uncertain, and sustained human-to-human transmission has not been demonstrated (Shaw et al., 2004; Mungmunpunipantip and Wiwanitkit, 2023; Zhang et al., 2025). For NiV/HeV, in contrast, Gates 1–2 are well supported, and Gate 3 varies by setting, making outbreak risk a function of both biology and context (Ambat et al., 2019; Monath et al., 2022). The framework helps avoid a common pitfall: equating molecular severity potential (Gate 2) with outbreak potential (Gate 3) (Meier et al., 2024). Instead, it clarifies what evidence would increase risk at each stage—for example, the demonstration of efficient respiratory shedding or repeated nosocomial chains would increase Gate 3 even if upstream exposure appears unchanged (Guyatt et al., 2008; Peel et al., 2019; Li et al., 2024; European Centre for Disease Prevention and Control, 2026).

Because the gates are separable, prevention can be aligned to the most leverageable bottleneck: reduce exposure (Step 0), block entry (Gate 1), blunt within-host fitness (Gate 2), or disrupt amplification networks (Gate 3) (Eaton et al., 2006; Halpin and Rota, 2015; Weatherman et al., 2018). This connects mechanistic biology to One Health intervention planning, rather than treating interventions as an afterthought (Khan et al., 2012; Dunga et al., 2025).

In the sections below, we apply the Confirmed/Supported/Unknown tags gate by gate, using Figures 1–4 and Tables 1, 2 to keep each claim tied to a specific evidence domain. Where evidence differs by strain or setting (e.g., NiV Bangladesh vs. Malaysia), we focus on the gate that changes rather than averaging across contexts (Satter et al., 2025). Rather than proposing universal numeric thresholds, we define gate-specific operational signals: receptor identification and entry for Gate 1; IFN antagonism, replication, and shedding-relevant tropism for Gate 2; and secondary transmission, epidemiologically relevant shedding, or amplifier-host involvement for Gate 3.

For NiV and HeV, Gate 1 is Confirmed: attachment and fusion glycoproteins enable entry via ephrin-B2/B3, which is consistent with a broad host range and access to key cell types once exposure occurs (Halpin et al., 2021; Carey et al., 2025; Shafaati and Zandi, 2025; Ahamed et al., 2026). For LayV and MojV, Gate 1 remains the dominant uncertainty (Chavda et al., 2023; Wang C. et al., 2024; Li Y. et al., 2025; Salleh, 2025). Current data support the nonuse of ephrin-B2/B3, but the true receptor(s), tissue permissiveness, and portal-of-entry constraints remain Unknown, limiting confident inference about human infection probability (Wang et al., 2000; Xu et al., 2008, 2012b; Chakraborty et al., 2022). Practical implications: a preparedness-focused synthesis should go beyond listing candidate receptors (Isaacs et al., 2023; May et al., 2023; Guo et al., 2024; May and Acharya, 2024). It should map likely sites of first replication (upper vs. lower respiratory tract, oral/GI mucosa, endothelium) and specify experiments that would resolve the uncertainty (binding assays, pseudo type entry across primary human cell panels, and *in vivo* entry-site models) (Choi, 2021). Link to figures: Exposure routes are summarized, and their translation into target-cell access at Gate 1 is mapped, underscoring that receptor biology connects ecology and mechanistic infection probability rather than serving as an isolated molecular detail (Figures 3, 4) (Lee et al., 2015; Oguntuyo et al., 2024; Navaratnarajah and Cattaneo, 2025).

Gate 2 integrates innate immune antagonism, replication competence, and tropism—together determining disease potential and the involvement of shedding-relevant tissues (McLinton et al., 2017; Devnath et al., 2022; Hu et al., 2025). For NiV and HeV, this gate is largely Confirmed: P-gene products (e.g., V/W/C proteins) antagonize interferon pathways and support systemic infection with neuro-respiratory involvement, which is consistent with severe outcomes in humans and multiple animal models (Geisbert et al., 2021; Madhukalya et al., 2025). For LayV and MojV, Gate 2 is situated between Supported and Unknown: genomic features suggest paramyxovirus-like immune evasion strategies, and some *in vitro* data indicate replication in selected systems, but comparative data on the strength of human innate antagonism, tissue tropism, and *in vivo* fitness remain incomplete (Rodriguez et al., 2003; Chen et al., 2020). This is where overinterpretation is most likely: sequence similarity cannot substitute for demonstrated IFN antagonism magnitude or tropism breadth (Cheliout Da Silva et al., 2021; Hejran et al., 2025). Practical implication: Gate 2 evidence should be reported as measurable outputs (IFN induction/antagonism metrics, growth kinetics, primary-cell replication, and organoid or animal-model tropism) and linked to downstream risk only through Gate 3 context—not treated as a proxy for outbreak potential (Monath et al., 2022; Mortlock et al., 2025).

Gate 1–2 synthesis should generate directly usable outputs: a receptor and entry matrix across human and target tissues in humans and amplifier hosts, a prioritized list of key unknowns, and standardized assays such as pseudotype entry panels, quantitative IFN antagonism readouts, and primary airway cell replication models (Rissanen et al., 2017; Navaratnarajah et al., 2020; He et al., 2024). These outputs should link portal of entry, first replication site, and clinically-relevant tropism, so that mechanistic findings can be interpreted as preparedness signals rather than as isolated molecular descriptors (Eaton et al., 2006; Halpin and Rota, 2015; Weatherman et al., 2018; Li et al., 2024; Rahim et al., 2024; Thien et al., 2024).

Gate 3 is where emergence risk becomes public health impact: amplification depends on shedding, contact networks, and often amplifier hosts that convert sporadic infection into clusters (Bhowmik et al., 2025). For NiV, Gate 3 is Confirmed but highly context dependent; outbreaks have included primary zoonotic cases as well as secondary person-to-person transmission, with healthcare and household close-contact networks repeatedly implicated (Branda et al., 2025; Satter et al., 2025; Ali et al., 2026). In Malaysia/Singapore, pig amplification generated high-dose exposure networks and large case numbers without sustained human-to-human spread. This highlights a key principle of the framework: Gate 3 can open via animal amplification even when human-to-human transmission is limited; thus, animal health interventions may be decisive outbreak levers (Misako et al., 2010; Castonguay et al., 2025). In Bangladesh, repeated seasonal outbreaks linked to date palm sap exposure reveal how a stable ecological interface can repeatedly seed primary infections. In some events, subsequent healthcare- or household-associated transmission occurred, illustrating that interface frequency (Step 0) and network amplification (Gate 3) can covary yet remain analytically distinct (Luby et al., 2009; Khan et al., 2012; Satter et al., 2023). HeV shows a contrasting Gate 3 pattern: spillover is strongly mediated by horses, and onward human-to-human transmission has not been a prominent feature. Amplification risk is therefore concentrated in the horse-care setting rather than broader community networks, supporting the interpretation that HeV Gate 3 is mainly driven by animal amplification (Halpin and Rota, 2015; Halpin et al., 2021).

For LayV, available evidence supports human infection in reported cases, but sustained human-to-human transmission has not been demonstrated. Gate 3 should therefore be treated as Supported or Unknown depending on the dataset and follow-up, and the most informative next evidence would come from rigorous cluster investigations, serological studies in contacts, and longitudinal viral shedding data (Hejran et al., 2025; Shafaati and Zandi, 2025). For MojV, the Gate 3 question is even further upstream: without confirmed human infection and validated entry or tropism mapping, any claim about transmission chains is premature. The framework helps prevent risk inflation by clearly separating plausible exposure from confirmed transmission (Rissanen et al., 2017).

Gate 3 cannot be inferred from within-host severity or neurotropism alone (Meier et al., 2024). It depends on shedding route and timing, contact-network structure, healthcare or household exposure, and animal amplification ecology (Halpin and Rota, 2015; Weatherman et al., 2018; Van Doremalen et al., 2022; Li W. et al., 2025). From a risk-gate perspective, IPC is a Gate 3 intervention that can close amplification even when Gates 1–2 are permissive (Sah et al., 2023). Importation is a special Gate 3 scenario: travel can introduce infection into new regions, but onward transmission depends on suitable reservoirs or amplifier hosts and on clinical detection and IPC. The absence of *Pteropus* fruit bats in Europe, for example, reshapes post-importation amplification even if a viremic traveler arrives (Madera et al., 2022; Monath et al., 2022).

Gate 3 evidence is most useful when summarized around four elements: (i) secondary attack patterns, (ii) transmission settings (household vs. healthcare), (iii) plausibility of aerosol/respiratory routes, and (iv) animal amplification pathways (Gurley et al., 2007; Mortlock et al., 2025). Consistent reporting of these elements consistently supports comparisons across outbreaks and helps define thresholds for escalating response (Ambat et al., 2019; Hassan et al., 2023; Qiu et al., 2024). Gate 3 is also where countermeasures most directly change trajectories: rapid diagnostics, IPC, ring vaccination (when available), and targeted prophylaxis can reduce effective transmission even when spillover continues (Hassan et al., 2025; Wu et al., 2025). Accordingly, Gate 3 synthesis should conclude with setting-specific intervention levers, rather than biological descriptors alone (Luby et al., 2009; Pollak et al., 2023; Satter et al., 2023). Together, these observations show that large human impact requires Gate 3 amplification to align with Gate 1–2 permissiveness within real contact networks (World Health Organization, 2022a; He et al., 2024; Li J. D. et al., 2025).

Gate-oriented outputs convert the framework into practical checklists for laboratories, surveillance teams, and outbreak responders (Table 1) (Annand et al., 2022; Rahim et al., 2024). Deliverable 1—Evidence-tagged transmission map: summarize confirmed, supported, and unknown evidence for human-to-human transmission, specifying the settings in which transmission was observed and the plausible exposure routes (Rahalkar and Bahulikar, 2020; Sun et al., 2025). This avoids collapsing heterogeneous outbreaks into a single averaged statement (Prescott et al., 2015; Wang Y. et al., 2024). Deliverable 2—Amplifier host pathways: state whether amplification is primarily driven by animal amplifiers (e.g., pigs, horses) or by human transmission chains and identify the ecological or occupational interfaces that enable it (Meier et al., 2024; Sun et al., 2024; Mortlock et al., 2025; Hantabal et al., 2026). This guides One Health prioritization (animal vaccination vs. hospital IPC vs. behavior change) (Castonguay et al., 2025; McLean et al., 2025). Deliverable 3—Shedding and timing: report what is known about shedding compartments and timing relative to symptoms because these determine whether syndromic surveillance and IPC are likely to interrupt chains (Paliwal et al., 2024; Wang Z. et al., 2024; Bhowmik et al., 2025). Where evidence is missing, state the gap explicitly and propose standardized sampling (Yadav et al., 2022; Satter et al., 2023; Dunga et al., 2025). Deliverable 4—Risk escalation triggers: list specific observations that would increase the risk at Gate 3 (e.g., repeated nosocomial clusters; evidence of respiratory shedding compatible with casual-contact spread) and those that would decrease it (e.g., negative contact testing across multiple events) (Luby et al., 2009; Peel et al., 2019). Deliverable 5—Strain and setting annotation: document how strain variation and healthcare capacity may modulate Gate 3 outcomes (case detection, PPE availability, crowding), without assuming that genetic differences alone explain outbreak differences (Hassan et al., 2023; Castonguay et al., 2025). Deliverable 6—Preparedness outputs: connect Gate 3 assessment to actionable response packages—diagnostic deployment, IPC escalation thresholds, contact-tracing depth, and criteria for when to consider vaccination or prophylaxis, if such tools exist (Monath et al., 2022; Satter et al., 2023). Deliverable 7—Cross-gate linkage: state explicitly which Gate 1–2 unknowns most limit Gate 3 inference for LayV/MojV (receptor identity, airway replication, shedding potential). This prevents inappropriate extrapolation from ecological detection alone (Frutos et al., 2022). Deliverable 8—Consistency between tables and figures: ensure that the narrative links Figures 1–4 and Table 2 such that geography (Figure 1) and interfaces (Figure 3) remain connected to mechanistic gates (Figure 4). To make the framework more operational, gate-specific evidence status, key uncertainties, and review-oriented outputs across the four representative viruses are summarized in Table 1.

**Box (current event): India 2026 Nipah through the risk-gate lens**

Event summary (official reporting): India has reported recurrent NiV outbreaks since 2018, most prominently in Kerala; therefore, the 2026 West Bengal event is used here as a focused application to a current event rather than as a comprehensive account of Indian NiV epidemiology. In early 2026, two laboratory-confirmed NiV cases were reported in West Bengal, India. Both were described as healthcare workers linked to a single hospital setting, and investigations focused on source identification and prevention of further transmission (Australian Centre for Disease Control, 2026; European Centre for Disease Prevention and Control, 2026; Press Information Bureau, Government of India, 2026; World Health Organization, 2026a).

Spatial and temporal context: The West Bengal cluster occurred in Barasat, North 24 Parganas district, near the Bangladesh border region where NiV outbreaks have recurred. A separate fatal case was reported shortly afterward in Naogaon District, Rajshahi Division, Bangladesh, approximately 250 km from Barasat, and was linked to repeated raw date palm sap consumption. Available official reports did not identify an epidemiological link between the West Bengal and Bangladesh events, supporting the interpretation that they were temporally and geographically proximate but epidemiologically separate events. The strain/genotype responsible for the 2026 West Bengal cluster was not specified at the time of revision, so no lineage-based inference is made here.

Gate 3 lens—amplification status: Contact tracing and testing revealed no additional positive cases among traced contacts at the time of reporting (the WHO reported >190 contacts testing negative; ECDC and Indian official statements described 196 contacts identified/tested and negative). This pattern is consistent with no detected expansion beyond the healthcare-linked cluster at the time of reporting, rather than evidence of wider community transmission (Kulkarni et al., 2013; Yadav et al., 2022; Rahim et al., 2024; Sahay et al., 2025). Gate-by-gate interpretation: Gates 1–2 for NiV are well supported (ephrin-B2/B3 entry and strong innate immune antagonism), so the immediate uncertainty is not whether NiV can infect humans but whether the amplification gate is opening under local contact patterns and clinical care conditions. The decisive levers are rapid detection, rigorous IPC, and interruption of contact networks (Xu et al., 2008; Australian Centre for Disease Control, 2026). Risk communication and importation: ECDC assessed the risk for Europeans traveling to or residing in the affected area as very low based on available information and noted that the absence of *Pteropus* fruit bats in Europe reduces the risk of onward transmission following a potential importation in the current context. This illustrates how reservoir ecology can shape post-importation Gate 3 risk. Framework takeaway: The same virus can be a high-consequence pathogen at Gates 1–2 yet manifest as a limited event at Gate 3, depending on the setting and response. The risk-gate lens makes this distinction explicit and helps avoid both complacency and overreaction.

## Preparedness implications of the risk-gate framework

Prevention should target the most leverageable bottleneck. Step 0 interventions reduce exposure (food safety around bat-contaminated products, animal husbandry changes, and interface risk reduction), whereas Gate 3 interventions reduce amplification (IPC, rapid diagnostics, contact tracing, and risk communication). These measures remain essential even as biomedical tools progress (Hassan et al., 2023; Sah et al., 2023; McLean et al., 2025). Biomedical countermeasures are moving forward (Playford et al., 2020; Román et al., 2022; Woolsey et al., 2022; Zhou et al., 2024). The University of Oxford reported the first Phase I (first-in-human) clinical trial of the ChAdOx1 Nipah B vaccine, which enrolled 51 adults aged 18–55, marking a shift from conceptual preparedness to defined development timelines (Foster et al., 2022; Chen et al., 2025b). This progress strengthens the rationale for gate-based deployment concepts—for example, where vaccination would most reduce Gate 3 amplification if efficacy is demonstrated (Chan et al., 2024; Rodrigue et al., 2024; University of Oxford, 2024; Lv et al., 2025). Governance and operational tools matter as much as products themselves (Bruno et al., 2022). The Quadripartite One Health Joint Plan of Action (2022–2026) provides a coordination framework across FAO/UNEP/WOAH/WHO, while the Tripartite Zoonoses Guide offers practical tools (e.g., joint risk assessment) for translating evidence into multisectoral action. We map these instruments to specific gates to make One Health implementation concrete rather than aspirational (Taylor et al., 2022; World Health Organization, 2022a, 2026b; Dunga et al., 2025).

## Discussion

Henipavirus emergence risk cannot be reduced to spillover frequency alone. A gate-based view clarifies why upstream exposure is necessary but insufficient and why outbreak impact depends on the alignment of mechanistic compatibility with real-world amplification. Across NiV and HeV, Gates 1–2 are broadly permissive; public health impact is often driven by the context of Gate 3—specifically amplification hosts, healthcare settings, and response speed. This implies that meaningful near-term risk reduction is achievable through targeted One Health and IPC investments even before next-generation biomedical tools mature. LayV and MojV show how uncertainty should be handled: their preparedness value lies in exposing unresolved gates, especially receptor identity, tissue permissiveness, and human infection or transmission status, rather than serving as direct analogs of NiV or HeV. We therefore distinguish missing data, mixed evidence, and the absence of documented transmission when assigning gate status. An important limitation is that this Hypothesis & Theory framework remains conceptual rather than formally validated; at this stage, its value lies in evidence mapping, comparative interpretation, and the generation of testable hypotheses. The framework is dynamic rather than static. Viral evolution may alter receptor use, tropism, shedding, or immune evasion, but sequence change alone does not establish altered emergence potential.

Future research should be explicitly mapped to gates: receptor discovery and entry-site models (Gate 1), standardized innate antagonism and tropism assays in relevant primary tissues (Gate 2), and longitudinal shedding and contact-network studies coupled with rigorous cluster investigations (Gate 3). Future work should test the framework against retrospective outbreak datasets, refine gate-specific scoring, and develop more objective criteria for gate transitions. Preparedness outputs should remain practical, including evidence-tagged risk statements, surveillance and IPC trigger lists, and gate-specific countermeasure deployment concepts; Table 1 provides a template for these outputs.

In summary, the risk-gate mechanistic–ecological framework links exposure, entry, and amplification in an actionable way for both science and policy. By tagging uncertainty and aligning interventions to gates, it supports more rigorous interpretation and more practical readiness for future henipavirus events.

## Data Availability

The original contributions presented in the study are included in the article/supplementary material, further inquiries can be directed to the corresponding authors.
